# Evaluating a LARC Expansion Program in 14 Sub-Saharan African Countries: A Service Delivery Model for Meeting FP2020 Goals

**DOI:** 10.1007/s10995-016-2014-0

**Published:** 2016-05-06

**Authors:** Thoai D. Ngo, Olivia Nuccio, Shreya K. Pereira, Katharine Footman, Kate Reiss

**Affiliations:** 1 Research and Knowledge Management, Innovations for Poverty Action, New Haven, CT USA; 2 Research, Monitoring and Evaluation Team, Health System Department, Marie Stopes International, 1 Conway Street, London, W1T 6LP UK; 3 Department of Global Health and Development, Faculty of Public Health and Policy, London School of Hygiene and Tropical Medicine, London, UK

**Keywords:** Sub-Saharan Africa, Family planning, Long-acting reversible contraception, Unmet need

## Abstract

*Objectives* In many sub-Saharan African countries, the use of long-acting reversible contraceptives (LARCs) is low while unmet need for family planning (FP) remains high. We evaluated the effectiveness of a LARC access expansion initiative in reaching young, less educated, poor, and rural women. *Methods* Starting in 2008, Marie Stopes International (MSI) has implemented a cross-country expansion intervention to increase access to LARCs through static clinics, mobile outreach units, and social franchising of private sector providers. We analyzed routine service statistics for 2008–2014 and 2014 client exit interview data. Indicators of effectiveness were the number of LARCs provided and the percentages of LARC clients who had not used a modern contraceptive in the last 3 months (“adopters”); switched from a short-term contraceptive to a LARC (“switchers”); were aged <25; lived in extreme poverty; had not completed primary school; lived in rural areas; and reported satisfaction with their overall experience at the facility/site. *Results* Our annual LARC service distribution increased 1037 % (from 149,881 to over 1.7 million) over 2008–2014. Of 3816 LARC clients interviewed, 46 % were adopters and 46 % switchers; 37 % were aged 15–24, 42 % had not completed primary education, and 56 % lived in a rural location. Satisfaction with services received was rated 4.46 out of 5. *Conclusions* The effectiveness of the LARC expansion in these 14 sub-Saharan African FP programs demonstrates vast untapped potential for wider use of LARC methods, and suggests that this service delivery model is a plausible way to support FP 2020 goals of reaching those with an unmet need for FP.

## Significance

Introduction of both contraceptive implants and intra-uterine devices is critical for reaching goals set out by the FP2020 initiative, but neither implementation experiences nor evaluations of program effectiveness in reaching those with unmet need for family planning have been widely documented. In this paper, we provide a description of programmatic approaches taken to achieve long-acting reversible contraceptive service delivery at scale in sub-Saharan African countries and results of an evaluation of program effectiveness.

## Introduction

Over two hundred million women and girls in developing countries lack access to contraceptives; the Family Planning 2020 (FP2020) initiative aims to reach 120 million new users by 2020 [3, 20]. In sub-Saharan Africa, the region with the greatest unmet need for contraceptives, provision over the past 30 years has relied predominantly on short-term methods (condoms, pills and injectables) [26]. Despite being cost-effective and highly efficacious [22], long-acting reversible contraceptive (LARC) availability and use is low while unmet need for family planning (FP) remains high, particularly in rural and remote regions of sub-Saharan Africa [2, 21].

Population-based surveys indicate that the average unmet need for modern methods of contraception is 34 % in West Africa and 31 % in East and Southern Africa (settings with high total fertility rates) [23, 26], and only one in four women in Africa uses a modern method of contraception [18]. Unintended pregnancies are common; 44 % of pregnancies in Eastern Africa in 2012 were unintended, 55 % in Southern and 26 % in West Africa [19]. Access to FP services is particularly low among rural, less educated, and poorer women [23, 25, 26]. Availability, perceived costs, lack of provider skills, and misperceptions about modern contraceptives and their risks and benefits are barriers to uptake [8, 23, 27].

Intrauterine devices (IUDs) and implants give effective long-term protection against pregnancy, with between 0.05 and 0.8 % of women experiencing pregnancy failure during the first year of use [7]. LARC use is associated with high user satisfaction and convenience, and LARCs have low discontinuation rates compared to short-term methods [9]. The expansion of access to LARCs as part of the full range of FP methods, is a critical component of FP service delivery programs that aim to address high rates of unintended pregnancy and curb high unmet need for FP.

Previous studies have shown success in expanding access to IUDs in sub-Saharan Africa, Latin America and Asia, with younger and less educated women reached through demand generation approaches and complementary service delivery mechanisms [1, 11]. Introduction of both implants and IUDs is critical for reaching goals set out by the FP2020 initiative to increase contraceptive prevalence, reduce unmet need and expand method mix. However, neither implementation experiences nor evaluations of program effectiveness in reaching those with unmet need for FP have been widely documented. In this paper, we describe the effectiveness of Marie Stopes International’s (MSI’s) integrated service delivery intervention designed to expand access to and knowledge of LARCs among women living in 14 sub-Saharan Africa countries.

## Methods

We conducted an evaluation of MSI’s LARC expansion intervention in 14 sub-Saharan African countries (Burkina Faso, Ethiopia, Ghana, Kenya, Madagascar, Malawi, Mali, Nigeria, Senegal, Sierra Leone, Tanzania, Uganda, Zambia and Zimbabwe).

We defined LARC users as women who chose IUD or implant services when presenting at an MSI service delivery site in the study countries.

### MSI’s LARC Expansion Intervention

This multi-country expansion intervention employed an integrated service delivery approach by expanding a network of providers in urban, peri-urban, and rural settings through four service delivery channels, coupled with a wide range of demand creation activities. Approaches are described below and in Table 1. Further detail on the approach and experiences of implementation can be found in a previously published article [10].

**Table 1:** Service delivery approaches used in the MSI LARC expansion program

Channel/intervention	Description
Static clinic	Located in urban areas Competitively priced Income generated from clinics located in wealthier areas subsidizes clinics in urban slums and/or mobile outreach units Serves as a base for training and logistics to support mobile outreach units, social franchisees, and community-based distributors
Mobile outreach	Visit communities with limited access to modern FP methods, such as rural villages and urban slums Provide free or subsidized FP services Pre-visit demand generation activities are conducted in partnership with local health care workers Typical team includes mid-level healthcare providers Operate from tents, vehicles or at government buildings Team may visit government clinics and complement the short-term methods provided
Social franchising of private sector providers	Partner with existing private health care providers, predominantly in small towns and urban/peri-urban slums Enables rapid scale up of access to quality FP and reproductive health services
Marie Stopes Ladies	Semi-urban areas Trained nurses and midwives working in the community to provide LARCs Data only included for Madagascar
Task sharing	Non-physician providers trained to provide clinical procedures otherwise restricted to higher level cadres, for example insertion and removal of implants and IUDs by nurses and midwives Enabled MSI to increase the number of service providers and FP procedures in rural and peri-urban areas
Alternative financing mechanisms	Aims to increase access to FP among clients who may otherwise be unable to afford services Paper or electronic vouchers (sent via SMS) for a particular service or range of services Public–private partnership: contracting local government authority outreach provision Working with health insurance schemes and development of community-based health insurance schemes
Demand generation	Use of local media; e.g., radio spots Education and awareness raising through community health workers and satisfied clients Roadshows Paper-based flyers and posters

#### Service Delivery Approaches

LARCs were delivered through MSI’s three main service delivery channels: static clinics, mobile outreach units, and social franchising of private sector providers. One program utilized a fourth mechanism of service delivery (Marie Stopes Ladies), where nurses and midwives provide services in rural and peri-urban communities as described in Table 1. In all channels, providers counselled on and offered short term methods (including condoms), LARCs and, where available, permanent methods, to ensure that women could choose the method that best suited their lifestyle, their fertility intentions and their contraceptive preferences. Comprehensive integrated family planning counselling was provided to all clients. This included client-centred two-way communication and active listening; assessing life style preferences and circumstances; assessing knowledge and information gaps; provision of information about the client’s chosen method including the benefits, risks, complications, and associated side effects; method of use or procedural information and alternative options available; use of models, leaflets, flip charts and examples of contraceptive methods; counselling on dual protection and STI/HIV risk assessment; provision of referrals for needed sexual and reproductive health services; and information on what to do or where to go in case of problems.

#### Task Sharing Initiative

The sharing of tasks or clinical procedures such as contraceptive counseling, and insertion or removal of implants and IUDs between different cadres of non-physician providers was implemented across service delivery channels wherever both legal and feasible.

#### Demand Creation Approaches

The service delivery models integrate a wide range of demand creation approaches to ensure women are aware of the contraceptive choices available to them and able to access MSI LARC services. Such activities promote the entire range of contraceptives offered, but where knowledge of a particular method(s) is low, or where certain misperceptions (such as incorrect health impacts of contraception or fears of infertility when using reversible methods) are a barrier to uptake, the programs aim to address these information gaps. Misperceptions of contraception were also addressed during contraceptive counselling at service delivery sites. Strong relationships with communities were developed by working with religious leaders, use of community health workers and peer educators, and partnering with local health authorities and local government clinics [13–15]. Demand-side financing approaches such as vouchers (in Ethiopia, Kenya, Madagascar, Sierra Leone and Uganda) further increased accessibility of LARCs and a full range of other contraceptive services [5].

#### Ensuring Quality

MSI maintains the quality of services provided across the 14 programs through upholding minimum standards for service delivery – a range of clinical guidelines and standard operating procedures that all programs must adhere to. All service providers receive training on FP methods, which includes counseling and choice of methods, and receive supportive supervision. All outlets (clinic, mobile outreach unit, social franchisee, Marie Stopes ladies) receive an internal audit at least annually, and each year randomly selected outlets receive a quality technical assurance (QTA) visit conducted by a medical advisor external to the program. Where necessary, QTA visits are followed-up with additional training, supportive supervision and further visits. Some programs undertake mystery client studies (also known as simulated patient studies) to better understand provision practices from the client perspective. In addition to the above, social franchisees are required to meet minimum standards before being signed-up, and must maintain those standards. These activities are rolled-out with the objective of ensuring the maximum possible clinical effectiveness, client safety and a positive client experience.

### Study Methods and Procedures

This evaluation used two sources of data: routinely collected health management information system (HMIS) and client exit interviews. Routine service data from 2008 to 2014 for the 14 country programs were collected and analyzed. Service data were collected via a paper-based or electronic HMIS. Where possible, data were collected at the client level; otherwise, service-level or aggregate-level data were collected for each facility or team providing services. Data were collated monthly and reported to a country-level central support office in aggregate form for quality checks. Any inconsistencies in data reporting were resolved by the country-level support office. Subsequently, data were sent to MSI’s central support office for further quality checks, and data discrepancies were resolved between the two levels of support offices.

Client exit interviews were conducted between April and December 2014, with the majority conducted between September and December 2014. When a country had 40 or fewer facilities or service delivery sites in the service delivery channel, all were included in the sample. When there were more than 40 facilities/sites, it was not considered feasible to visit all facilities/sites, so a systematic sample of sites were taken. A skip pattern was determined based on the number of facilities/sites in the service delivery channel, and the skip pattern was used to select sites from a list which had been ordered by the average daily number of clients to ensure that facilities/sites of a variety of sites were selected. At the facility/site, a skip pattern was used to systematically select clients to be asked for an interview, for a set number of days per facility/site. The skip pattern was determined based on the average number of client visits per day at that site, to ensure that clients were selected across different times of day. More interviews were conducted at facilities/sites with a higher client flow due to the use of a standardized skip pattern and the set number of days per facility/site. The minimum sample size was determined for each service delivery channel in each country to ensure a representative sample of clients for the period of data collection providing indicators with 95 % confidence intervals of not more than ±10 %. The sample size was calculated for a hypothetical key indicator with coverage of 50 % for a conservative sample size estimate. In service delivery channels where not all facilities/sites were included, the sample size was doubled to account for the design effect of clustering by facility/site. The minimum sample size was also increased by 10 % to allow for non-response, giving a minimum sample size of 107 in service delivery channels where all facilities/sites were sampled, and 214 in sites where a selection of sites were sampled.

Clients were interviewed after receiving a service from MSI using a standardized questionnaire, and were interviewed one-on-one by trained research assistants. Clients were asked about their socio-demographic characteristics, contraceptive behaviours, and satisfaction with any services received. All clients provided informed consent prior to the interview. The average response rate was 96 % across 8 countries that collected response rate information, and ranged from 79 to 99 %. Response rates were not adequately reported from 6 of the programs. Data were entered into standard data entry forms using Epi Info, and went through two rounds of data cleaning (in country and at head office) to assess and ensure data quality. MSI’s independent ethics review committee approved the client exit interview protocol. Ethical clearance was not sought for the collection of routine HMIS data as only aggregate data were available from the global system used to extract data (e.g. number of implants provided in country X in year X), and no client-level information is available in this system.

The percentage of clients who were “adopters” (women who had not used a modern method of family planning in the last 3 months) and “switchers” (women who switched from a short-term contraceptive method to a LARC) were used as proxy indicators of the program’s effectiveness in addressing unmet need for long-acting contraception. “First-time users” is the commonly used metric by which programs have documented their success in reaching additional women with FP services; however, this measure underestimates the extent to which a program is reaching women with unmet need. The “adopter” metric was developed to better capture this group; as well as first-time users, it includes women who might have used contraception previously but are currently at risk of unintended pregnancy [17].

Research staff familiar with the local contexts in each country classified the location where clients received services as rural, urban or peri-urban, except in Zimbabwe, where location was not recorded. The poverty status of clients was assessed using the Progress Out of Poverty Index (PPI) in all countries except Zimbabwe and Madagascar, where the multidimensional poverty index (MPI) was used [6, 16]. Clients were considered to be living in extreme poverty if living on less than purchasing-power parity-adjusted $1.25 a day (according to the PPI) or multidimensionally poor (according to the MPI). As part of the client exit interview survey, clients were asked to use a Likert scale to rate various aspects of service delivery, including waiting times, length of time with a health care provider, quality of advice and information, and friendliness and respect from staff. Scores of 1 (indicating very bad) to 5 (indicating very good) were given to each aspect. There were low levels of missing data across almost all variables, ranging from 0.24 % missing marital status to 1.13 % missing age, and indicators were calculated using only available data. Missing data was higher for the PPI indicator (3.57 %), as only respondents who answered all ten PPI questions should be included in the overall score [6].

To allow comparison to national figures, the proportions of our participants who were aged less than 25 years, had not completed primary education, and lived in extreme poverty were compared to regional data from Demographic Health Surveys (DHS) and the World Bank PovCalNet website.

Service statistics data from HMIS were stored in Microsoft Excel 2011^®^. All exit interview data were analyzed in STATA version 13 (College Station, Texas, USA). Exit interview data were summarized using descriptive analyses. Differences in socio-demographic and reproductive characteristics between IUD and implant users were assessed using Chi squared tests and t-tests. Data were adjusted during analysis for the sampling design effect.

All the exit interview results presented are weighted by client flow; that is, the number of LARC clients that came through each channel and each country program in 2014.

## Findings

### Service Provision of LARC in 14 Countries

Between January and December 2014, 1,703,576 LARC services were delivered (1,334,566 implants and 369,010 IUDs) through the 14 sub-Saharan Africa programs. From 2008 to 2014, uptake of IUDs increased by 429 % and uptake of implants by 1567 % (Table 2). The number of LARC services delivered in 2014 was over ten times that in 2008 (an increase of 1037 %).

**Table 2:** LARC provision, 2008–2014, overall and by region

Region	2008	2009	2010	2011	2012	2013	2014
*Implants*							
East and Southern Africa	77,409	171,422	241,431	359,503	625,603	750,458	1,018,360
West Africa	2632	20,855	49,276	75,348	127,588	217,343	316,206
Total implants	80,041	192,277	290,707	434,851	753,191	967,801	1,334,566
*IUDs*							
East and Southern Africa	66,354	83,316	138,744	141,633	183,257	219,557	285,181
West Africa	3486	11,518	21,364	27,741	43,301	76,061	83,829
Total IUDs	69,840	94,834	160,108	169,374	226,558	295,618	369,010
*Total LARCs*							
East and Southern Africa	143,763	254,738	380,175	501,136	808,860	970,015	1,303,541
West Africa	6118	32,373	70,640	103,089	170,889	293,404	400,035
Total	149,881	287,111	450,815	604,225	979,749	1,263,419	1,703,576

MSI programs in East and Southern Africa drove most of the total growth in LARC services expansion, with 1,303,541 services delivered in 2014. Nevertheless, LARC services delivered in the West African programs included in this evaluation, which are fewer in number, had high growth between 2008 and 2014—with LARC services growing 6439 % in 2008–2014—but made-up a smaller share of total LARC services. In 2014, 24 % of all our LARC services were delivered by the six included West African programs (Burkina Faso, Ghana, Mali, Nigeria, Senegal, and Sierra Leone), while the remaining 76 % of services were delivered by programs in East and Southern Africa.

### Socio-Demographic Characteristics of LARC Clients

In total, 3816 LARC users (3214 implant users, and 602 IUD users) participated in exit interviews after receiving services from static clinics, mobile outreach units, social franchises and Marie Stopes ladies in the 14 national programs. Thirty-seven per cent were aged 15–24, 42 % had not completed primary education, 84 % were married or living with a partner, and 56 % lived in rural locations. We observed some notable differences between IUD and implant users. IUD users were older (28 % aged over 35 years compared to 15 % among implant users, *P* < 0.01) and more educated (24 % had completed secondary education or above compared to 16 % of implant users, *P* = 0.03). IUD users were also more likely to live in an urban location (41 % compared to 28 % of implant clients, *P* < 0.01). The two groups of users had similar marital status and parity (Table 3).

**Table 3:** LARC user profile, by contraceptive type and region

	All countries				East and Southern Africa				West Africa			
					Ethiopia, Kenya, Madagascar, Malawi, Tanzania, Uganda, Zambia, Zimbabwe				Burkina Faso, Ghana, Mali, Nigeria, Senegal, Sierra Leone			
	LARC users % (n = 3816)	Implant users % (n = 3214)	IUD users % (n = 602)	*P* value	LARC users % (n = 1831)	Implant users % (n = 1548)	IUD users % (n = 283)	*P* value	LARC users % (n = 1985)	Implant users % (n = 1666)	IUD users % (n = 319)	*P* value
*Age group*												
15–24	37	39	21	<0.01	39	42	24	<0.01	29	32	10	<0.01
25–34	45	44	50		45	44	53		45	46	40	
35+	17	15	28		15	14	23		24	20	49	
Unknown	1	1	0		1	1	0		1	1	1	
*Parity*												
None	8	8	10	0.06	9	8	12	0.122	6	6	2	<0.01
1–2	42	43	36		46	46	41		31	33	17	
3–4	31	31	31		31	31	31		31	31	32	
5+	19	19	23		15	15	16		32	29	48	
Unknown	0	0	0		0	0	1		0	0	0	
*Education*												
None/some primary	42	43	40	0.03	38	39	34	0.05	55	54	62	0.01
Primary/some secondary	39	39	35		43	43	41		26	28	16	
Completed secondary	17	16	24		17	16	24		18	18	22	
Unknown	1	1	1		2	2	2		1	1	1	
*Marital status*												
Single	13	13	12	0.44	15	14	15	0.4	9	10	4	0.05
Married/living with partner	84	83	86		82	82	84		88	87	93	
Divorced/widowed/separated	3	3	2		3	4	2		2	2	3	
Declines to answer	0	0	0		0	0	0		0	0	0	
*Location type*												
Urban	30	28	41	<0.01	29	26	42	<0.01	34	34	38	0.07
Rural	56	58	44		58	61	45		50	51	41	
Peri-urban	14	14	15		13	13	13		16	15	21	

### Comparison to Demographic Health Survey Datasets

Over a third [37 %, 95 % CI 34–39) of interviewed LARC users were aged 15–24 years in 2014, ranging from 14 % in Nigeria to 52 % in Malawi, compared to a cross-country DHS average proportion of LARC users of 13 % (range: 2 % in Zambia to 27 % in Sierra Leone). Of MSI’s LARC users, 42 % did not have complete primary education (95 % CI 40–45), ranging from 0 % in Uganda to 84 % in Burkina Faso. This compares to a cross-country DHS average proportion of 45 % of LARC users (range: 5 % in Zimbabwe to 89 % in Ethiopia). World Bank poverty estimates could not be confined to LARC users, so we compared the poverty status of MSI clients to that of the entire national population. The proportion of poor within the MSI client population, 38 % (data not shown), was lower than the average of 49 % in the 13 countries with comparison data available (excluding Zimbabwe). However, 44 % of LARC users served through outreach (the service delivery channel that expands access to the most underserved populations) were poor, which is similar to the overall population average.

### Adoption of Contraception and Switching from Short-Term Methods

About half (46 %) of LARC users were adopters (women who had not used FP in the previous 3 months): 49 % at mobile outreach; 45 % in static clinics; 33 % in social franchises. About half (46 %) switched from a short-term method: 44 % at mobile outreach; 45 % in static clinics; 54 % in social franchisees (Table 4). The remaining 8 % of clients were continuing users of long-acting contraception.

**Table 4:** LARC users, contraceptive behaviours, overall and by region

Region	Overall				East and Southern Africa				West Africa			
	All countries				Ethiopia, Kenya, Madagascar, Malawi, Tanzania, Uganda, Zambia, Zimbabwe				Burkina Faso, Ghana, Mali, Nigeria, Senegal, Sierra Leone			
	LARC users % (n = 3816)	Implant users % (n = 3214)	IUD users % (n = 602)	*P* value	LARC users % (n = 1831)	Implant users % (n = 1548)	IUD users % (n = 283)	*P* value	LARC users % (n = 1985)	Implant users % (n = 1666)	IUD users % (n = 319)	*P* value
*Adopters*												
% of women who had not used FP in the previous 3 months	46	47	44	0.05	42	42	41	0.07	59	60	55	0.63
*Switchers*												
% of women who switched from a short-term to a long-term FP method	46	46	47	0.85	50	50	50	0.80	34	34	36	0.28

### Socio-Demographics of LARC Users by Service Delivery Channel

Table 5 contains data on reach of underserved groups by service delivery channel, excluding Marie Stopes Ladies, a channel only available for one country (Madagascar). Statistically significant differences were observed for variables related to poverty, education, and urban/rural location. Clients receiving a LARC method at outreach sites were more likely to be living in extreme poverty than at static clinics or social franchise clinics. Fifty-four per cent of social franchise clients had not completed primary education, compared to 44 % of outreach clients and 20 % of static clinic clients. Outreach is a predominantly rural service delivery channel, with 71 % of clients visiting sites in a rural location. Overall, similar proportions of LARC users were aged under 25 at all three service delivery channels, though static clinics and social franchise clinics had higher proportions of under-25 s than outreach sites in West Africa.

**Table 5:** Underserved clients by delivery channel, overall and by region

	All countries				East and Southern Africa				West Africa			
					Ethiopia, Kenya, Madagascar, Malawi, Tanzania, Uganda, Zambia, Zimbabwe				Burkina Faso, Ghana, Mali, Nigeria, Senegal, Sierra Leone			
	Outreach % (n = 2455)	Social Franchise Clinics % (n = 651)	Static clinics % (n = 587)	*P* value	Outreach % (n = 1172)	Social Franchise Clinics % (n = 292)	Static clinics % (n = 244)	*P* value	Outreach % (n = 1283)	Social Franchise Clinics % (n = 359)	Static clinics % (n = 343)	*P* value
*Age group*												
15-24	36	36	39	0.83	40	34	39	0.49	26	41	42	<0.01
*Extreme poverty*												
Living on <$1.25 a day or multidimensionally poor	44	30	13	<0.01	49	31	12	<0.01	30	27	18	0.89
*Education*												
Incomplete primary education	44	54	20	<0.01	40	54	18	<0.01	56	53	43	0.29
*Location*												
Rural	71	24	2	<0.01	77	24	2	<0.01	57	26	5	<0.01

### Acceptability of LARC/Services Among Women

High levels of client satisfaction were reported for LARC clients across all service delivery channels in 14 country programs. Over 99 % of LARC users indicated that they would use MSI services in the future and 99 % indicated that they would recommend MSI to a friend. The mean score for overall satisfaction with services received was 4.46 (95 % CI 4.40–4.51). Mean scores ranged from 4.16 (95 % CI 4.10–4.23) for waiting times to 4.50 (95 % CI 4.47–4.56) for friendliness and respect of the staff at the site or facility.

## Discussion

This paper describes the effectiveness of a LARC expansion intervention in 14 sub-Saharan African countries and demonstrates the untapped potential for wider use of LARCs across the region. Between 2008 and 2014, there was a 1037 % increase in the use of MSI LARC services across 14 countries in sub-Saharan Africa; from 149,881 services in 2008 to 1,703,576 in 2014. This LARC expansion initiative has been successful in expanding access to contraception to a diverse group of women with high unmet need for FP, through MSI’s three main service delivery channels (static clinics, outreach, and social franchise clinics), plus Marie Stopes Ladies in Madagascar. The majority of LARC provision was in East and Southern Africa, while West African provision was lower, possibly due to the more recent initiation of these programmes, lower levels of donor funding available, relatively smaller population sizes, and social desirability of many children [12]. Implant uptake was higher than IUD uptake, suggesting higher acceptability of the method which may be due to ease of insertion, method of insertion, length of efficacy, or preference for hormonal methods, but more research is needed to fully understand the variations in uptake.

Complementary service delivery channels enabled programs to expand access to LARCs to individuals across socio-demographic divides (Table 5) while serving those who previously had an unmet need for long-acting methods and increasing demand for LARCs. The service delivery sites attained high levels of client satisfaction. The results presented here demonstrate that mobile outreach services are reaching underserved populations in rural areas with quality services, and support the use of outreach services to overcome some of the structural barriers to effective and high quality service delivery which often exist in rural locations such as restricted mobility, poor-quality health facilities, lack of trained personnel, and inadequate information about contraceptive choices [24, 26]. Furthermore, 46 % of LARC users had not used a modern method of contraception over the previous 3 months, and 46 % switched from a short-term to a long-term contraceptive method at the time of visit, demonstrating that this initiative was able to expand the available method mix and address unmet need in this population. This initiative also reached a high proportion of younger women, especially in comparison with country-level data on method mix from the DHS; this is particularly noteworthy, as this group usually faces greater levels of unmet need.

MSI’s comprehensive service delivery model utilizing static clinics, mobile outreach units, and social franchises, and innovative approaches including alternative finance mechanisms (vouchers), demand generation in advance to outreach visits, and task sharing strategies in mobile outreach units, increased demand for and uptake of LARCs, especially among underserved populations. Programmatic evidence from initiatives in other countries has demonstrated similar success in increasing availability of LARC services. In 2003, PSI Nepal’s Sun Quality Health Network created a social franchise with private clinic partners across the country [4]. Through social franchised stationary and mobile clinics and health fairs to improve counseling for long-acting permanent methods, this initiative provided 2000 IUDs and 6000 sterilizations over a span of 3 years. Similarly, early public–private partnerships in Ghana and Tanzania resulted in significant increases in the number of IUDs and female sterilization services provided [4]. These examples show that improving access to contraceptive choices requires utilization of robust service delivery models that incorporate innovative delivery mechanisms, public–private partnerships, voucher schemes and task sharing, in order to reach those most at need for FP services.

### Limitations

As this was not an experimental study, we cannot separate the effects of various program expansion components on the results observed. We also cannot explore the cost-effectiveness of this initiative in this analysis, though this has been explored for expanding IUD provision in Africa in a previous publication [11].

Data for this study was extracted from exit interviews, which represent a cross-sectional sample of our clients, and therefore may not be representative of our entire client population across the year if there are seasonal differences in family planning uptake and clientele. Exit interview samples were calculated for MSI’s entire client base, and LARC users were not specifically sampled. Courtesy bias and the validity and reliability of the Likert scale are limitations associated with satisfaction data and the instrument used to collect this information. Satisfaction scores are generally high in exit interviews, and follow up studies may be needed to better access client experience. Classification of facilities and sites as urban or rural was completed by research staff in each country and was not standardized between countries, reducing our ability to make cross-country comparisons. Lastly, our analysis utilized poverty proportions for the overall population from the World Bank and compared these to the proportion of poor in our study population (LARC users). This comparison of country-representative samples in World Bank data to all MSI LARC users is not a like-with-like comparison and is a limitation of this analysis; further research is required to accurately determine effectiveness in reaching poorer populations.

### Recommendations

In order to increase women’s contraceptive choices in sub-Saharan Africa, and to expand access to quality LARC services, we recommend that FP organisations: (1) use complimentary service delivery channels to reach populations in varying locations and with different socio-economic profiles; (2) engage with private providers to build capacity to provide high-quality, client-focused LARC services; (3) expand availability of LARC services in rural areas, through the use of outreach services; (4) increase public awareness about the benefits of LARCs by employing method-specific marketing and interpersonal communications campaigns with a mixed-media approach; (5) ensure that task-sharing of clinical tasks and procedures follows WHO guidelines where legal and feasible; and (6) safeguard the quality of family planning service provision using minimum standards, provider training, monitoring and supportive supervision.

Family planning organisations must also address ethical issues by creating mechanisms that ensure a full choice of methods are available and encouraged and ensuring removal and re-insertion services continue to be available when methods expire or women decide to change method or choose to have children.
